# A Treatise on Albuminuria

**Published:** 1881-03

**Authors:** 


A Treatise on Albuminaria. By W. Howship Dickinson, M.D., Cantab, Fellow of the Royal College of Physicians, etc. Second edition. Wm. Wood & Co., New York. 1881.

These last two books are of the series of "Wood's Library of Standard Medical Authors," and come to us in the changed and improved garniture that has been adopted and inaugurated with these two volumes.

With reference to the work of Prof. Pifford his name is sufficient guarantee of the merit of the book.

The varied pathological conditions of the kidneys or of other organs that either directly or indirectly occasion albuminaria are complicated, and often are the source of much confusion and uncertainty to the practitioner. The work of Dr. Dickinson is proposed to elucidate the subject, and by a complete survey of the whole field, present a book of reference in easy reach of all. With the many splendid illustrations and the exhaustive discussion of each pathological condition the project of presenting a full and compendious treatise on the subject seems to be fully carried out.

